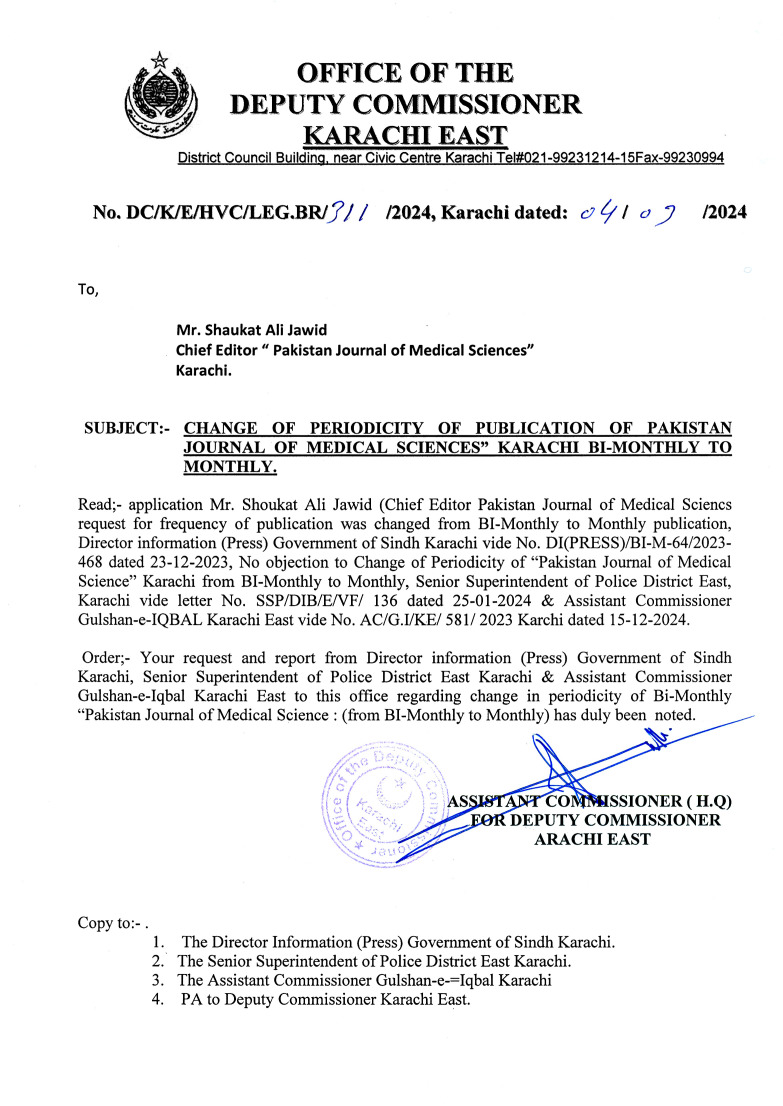# 

**Published:** 2024